# Gustatory Dysfunction among a Sample of Depressed Egyptian Adults under Antidepressants Therapy: A Retrospective Cohort Study

**DOI:** 10.1155/2021/5543840

**Published:** 2021-03-04

**Authors:** Christine Mikhail, Khaled Elgaaly, Ahmed Abd El Latif Abd El Hamid, Olfat Shaker, Shereen Ali

**Affiliations:** ^1^Oral Medicine and Periodontology Department, Faculty of Dentistry, Cairo University, Cairo, Egypt; ^2^Oral Medicine and Periodontology Department, Faculty of Dentistry, Fayoum University, Faiyum, Egypt; ^3^Department of Psychiatry, Faculty of Medicine, Cairo University, Cairo, Egypt; ^4^Medical Biochemistry and Molecular Biology Department, Faculty of Medicine, Cairo University, Cairo, Egypt

## Abstract

It is quite clear that the ability to perceive taste sensations significantly affects food choice, which consequently affects health status in the long term. Gustatory dysfunction is a neglected symptom among the depressed patients and those under antidepressants therapy, although these patients are suspectable to oral problems, due to general self-negligence related to mental disease, fear of dental treatment, and side effects of varied medications utilized in psychiatry. This study is aimed at assessing gustatory thresholds (detection and recognition thresholds) among a sample of 30 depressed Egyptian adults under antidepressants therapy for at least 3 months or psychotherapy with age ranging from 20 to 50 years old, seeking the Psychiatric Clinic at the Faculty of Medicine, Cairo University, Egypt. These patients were distributed into three equal groups (tricyclic antidepressants (TCA), selective serotonin reuptake inhibitors (SSRIs), and psychotherapy) and were assessed for gustatory detection and recognition thresholds using the filter paper disc method through a scoring system. The participants were also divided into normal taste group in which both the detection and recognition scores were 1, while the scores from 2 till 5 were considered as hypogeusia group and the score of 6 was considered as dysgeusia group. The TCA group was statistically significant from the other 2 groups in sweet detection thresholds (*p* = 0.043) and sweet recognition thresholds (*p* = 0.007). Hypogeusia for sweet was statistically significant (*p* = 0.041), where it was more common among TCA (70%) than both SSRIs and the psychotherapy group (20%). Gustatory dysfunction was found to be mostly associated with TCA followed by SSRIs particularly for sweet taste thresholds. More attention has to be given to taste changes among these patients as oral health affects general health by causing considerable pain and by changing what people eat, their speech, and their quality of life and wellbeing. Proper awareness and evaluation of this problem will improve the quality of life for the depressed patients and avoid unnecessary treatment. This trial is registered with ClinicalTrials.gov ID: NCT03599011.

## 1. Introduction

The depressed patients and those under antidepressants therapy are susceptible to oral problems, due to general self-negligence related to mental disease, fear of dental treatment, and side effects of varied medications utilized in psychiatry. Moreover, in recognition of a wide range of microbials, stress and damage signals, had been considered as the most important factors responsible for tissue damage linked with periodontitis [1].

In this regard, it has been hypothesized that conditions such as periodontitis and Coronary heart diseases may determine the increased release at the serum and salivary levels of IL-1, IL-6, prostaglandins, metalloproteases, NO, and hs-CRP, which, in turn, negatively influences the tone of the endothelial wall and, finally, causes a high risk of endothelial dysfunction and CHD development [2]. Oral and periodontal diseases can determine severe functional, phonatory and aesthetic impairments and are the main cause of adult tooth loss. They are more frequent in the disadvantaged segments of society and, in particular, in subjects who have difficulty accessing preventive services and dental care [3]. Also, the relationship between taste sensation, intake behavior, and long-term health outcomes is complex, but it is quite clear that the ability to perceive taste sensations significantly affects food choice, which consequently affects health status in the long term [4].

Taste changes can be classified into hypogeusia (diminished taste sensation), dysgeusia (taste distortion), phantogeusia (phantom taste), and ageusia (taste loss) [5]. Systemic conditions and their treatments are known to influence oral health (e.g., reduced saliva flow). Saliva provides protection of hard and soft tissues; aids taste, swallowing, and digestion; and offers antimicrobial properties. Saliva contains more than two thousand proteins, enzymes, electrolytes, small organic molecules, and antimicrobials; identification and use of these salivary biomarkers is ongoing for many conditions in research such as oral/oropharyngeal cancer [6].

Taste changes are often drug related. For example, in one study, for approximately 25% of patients with taste disturbances at an ear, nose, and throat outpatient clinic, the problem was drug related [7]. 12% of olfactory dysfunction may be attributed to medication effects, and an even larger proportion of gustatory dysfunction. However, conclusive data confirming the role of medications in smell and taste disorders are few, because most evidence is based on case studies or series [8].

Data on medical variables suggested that mood disorders ranked first while anxiety disorders ranked second, 34.6 and 27.8%, respectively, followed by psychoactive substance use disorders (21.1%) and schizophrenia (6.8%). It was also suggested that 39.8% of patients had a duration of mental illness of 1–5 years, 88% of patients were independents for their daily life activities, and 65.4% of patients had no healthy practices Shah et al. [9], Shukla and Srivastava [10] suggested the reverse findings, with anxiety disorders being on top of the list and mood disorders ranking second despite the low proportion of patients (i.e., 19 and 10%). Chandu et al. [11] had similar findings; they mentioned that 45% of patients were suffering from mood disorders followed by schizophrenia (25%) and psychoactive substance use disorders (20.6%).

In circumstances such as anxiety or depression, in which serotonin (5-HT) and noradrenaline (NA) are altered, they are associated with taste disturbances, indicating the necessity of these transmitters in the determination of taste thresholds in health and disease [12]. Most of the prescribed antidepressant agents are associated with a number of significant oral complications, including sialadenitis, xerostomia, gingivitis, glossitis, stomatitis, dysgeusia, tongue edema, and discoloration, which appear almost due to salivary gland dysfunction caused by the medication [13].

It has been reported clinically that both first-generation tricyclic antidepressants (TCA) and later-developed selective serotonin reuptake inhibitors (SSRIs) cause chemosensory complaints. Despite these helpful clinical observations in determining potential chemosensory side effects of drugs, it is essential to quantify the potential taste changes by carrying out experimental studies [14]. Oral pathologies are a public health problem that has a growing prevalence that, if not properly treated, can affect the relational, psychological, and social skills of an individual [15].

The rationale of conducting such study was to determine that gustatory thresholds (detection and recognition thresholds) among a sample of depressed Egyptian adults under antidepressants therapy (TCA and SSRIs) for at least 3 months were not affected as compared to those under nonpharmacological therapy (psychotherapy), seeking the Psychiatric Clinic at the Faculty of Medicine, Cairo University, Egypt.

## 2. Materials and Methods

STROBE statement checklist was followed in reporting cohort studies. This trial is registered with ClinicalTrials.gov ID: NCT03599011.

The study was approved by the Ethical Committee of the Faculty of Dentistry, Cairo University (Ethical Committee Approval Number 18936). The aim of the study and its benefits were explained to each participant with emphasis on confidentiality of the collected data. Each participant signed an informed consent before being enrolled in the study.

### 2.1. Study Design

This is a retrospective cohort study.

### 2.2. Setting

The subjects had been recruited from the Psychiatric Clinic at the Faculty of Medicine, Cairo University, Egypt.

### 2.3. Participants

A sample of 30 depressed patients under antidepressants therapy for at least 3 months or psychotherapy, with age ranging from 20 to 50 years old, seeking the Psychiatric Clinic at the Faculty of Medicine, Cairo University, was recruited during a period of almost 7 months starting from February 2020 to August 2020. Those who were of ages other than the mentioned, or under antipsychotics, hypnotics, or anticonvulsants therapies or suffered from olfactory dysfunction, were excluded.

The thirty eligible patients were recalled using their phone numbers recorded in their follow-up records, were asked to come to the Diagnosis Center at the Faculty of Dentistry, Cairo University, in order to be assessed for gustatory functions and were given identification numbers and distributed into three equal groups (Exposure 1: TCA; Exposure 2: SSRIs; and non-Exposure: psychotherapy), to which the operator was blinded to avoid selection bias. Nonrespondent bias was minimized by explaining to the participants the aim of the study and their importance and role in the study.

### 2.4. Variables and Data Sources/Measurement

#### 2.4.1. Outcomes

The gustatory thresholds were assessed using filter paper disc method in which test discs were placed on the tongue (Nagai et al. [16]). The gustatory tests were carried out in the morning during fasting state using five concentrations of the substances to test the four tastes: sweet, salty, sour, and bitter. Five concentrations of each of the four tastes were only used, as, practically, one should be cautious that subjects undergo sensory fatigability which causes limitations to the number of tests that can be done. So, when determining recognition thresholds, a number of five or six different compounds is the maximum recommendation to be investigated [17].

Test discs of 5 mm in diameter were wetted with a solution and placed on the left lateral part of the tongue at approximately 2 cm from the proglossis (the tip of the tongue), which is thought to be innervated by the chorda tympani nerve. During the tests, the participants were instructed to rinse their mouths with distilled water before testing the next concentration. A one-minute interval after water rinses is necessary before presenting further solutions.

The five concentrations of the four tastes (sweet, salty, sour, and bitter) used were starting from the lowest concentration then ascending to the highest possible concentration of each taste as follows: sweet—0.8 M, 1.2 M, 1.4 M, 1.6 M, and 2 M; salty—0.02 M, 0.1 M, 0.2 M, 0.3 M, and 1.2 M; sour—0.01 M, 0.02 M, 0.06 M, 0.1 M, and 1.2 M; and bitter—0.04 M, 0.15 M, 0.3 M, 0.6 M, and 0.9 M.

The concentrations of each taste were serially scored from disc number 1 (lowest) to number 5 (highest). If the subject was not able to recognize the taste at the highest concentration, a score of 6 was given. The detection threshold was defined as the concentration at which the subjects clearly indicated it as different from deionized water, but not necessarily recognizing the quality of the taste, while the recognition threshold was defined as the solution at which the subjects clearly identified the quality of the taste. The participants were also divided into normal taste group in which both the detection and the recognition scores were 1, while scores from 2 till 5 were considered as hypogeusia group and a score of 6 was considered as dysgeusia group.

#### 2.4.2. Exposures

  E1: Exposure 1—commonly prescribed tricyclic antidepressants (imipramine, amitriptyline, and clomipramine HCL)
  E2: Exposure 2—commonly prescribed selective serotonin reuptake inhibitors antidepressants (fluoxetine, fluvoxamine, sertraline, citalopram, and paroxetine)

#### 2.4.3. Nonexposure

This includes nonpharmacological treatment (psychotherapy).

#### 2.4.4. Predictors

Age ranged from 20 to 50 years old. Gender was assessed as male or female. Social level was assessed by residence in urban or rural areas. Time since starting antidepressants therapy: at least 3 months ago. Presence of complications associated with antidepressants therapy: gustatory dysfunction (taste changes) was assessed by taste intensity measurements.

#### 2.4.5. Effect Modifiers

The participant's mouth was rinsed with distilled water before testing the next concentration to avoid the affection of taste intensity measurements by the presence of residual stimuli from prior testing (Zverev [18]).

#### 2.4.6. Potential Confounders

It was quite clear that the ability to perceive taste sensations significantly affected food choice, which consequently affected health status in the long term (Snyder and Bartoshuk [4]).

#### 2.4.7. Bias

The eligible patients were recalled, given identification numbers and divided into three groups, to which the operator was blinded to avoid selection bias. Nonrespondent bias was minimized by explaining to the participants the aim of the study, their importance, and role in the study. Incomplete records were excluded from statistical analysis with reporting the cause of not completing the record.

#### 2.4.8. Sample Size

Based on the previous paper by Ramzy [19], taste disturbances were found in 8% in SSRIs and 83% in tricyclic group. Using power 95% and 5% significance level, 8 patients in each group were required. Sample size was calculated by PS program.

#### 2.4.9. Statistical Methods

All the results were subjected to statistical analysis. Data management and statistical analysis were performed using the Statistical Package for Social Sciences (SPSS) version 25. Numerical data were summarized using means and standard deviations and ranges. Categorical data were summarized as percentages. Data were explored for normality using Kolmogorov–Smirnov test and Shapiro–Wilk test. Comparisons between the 3 groups with respect to numeric variables were done using the one-way ANOVA. Pairwise differences were detected by Bonferroni's post hoc test. For categorical variables, differences were analyzed with chi square (*χ*^2^) test and Fisher's exact test when appropriate. Adjustments of *p* value were done using the Bonferroni method for multiple testing. All *p* values are two-sided. *p* values ≤ 0.05 were considered statistically significant.

## 3. Results

**Figure 1:**
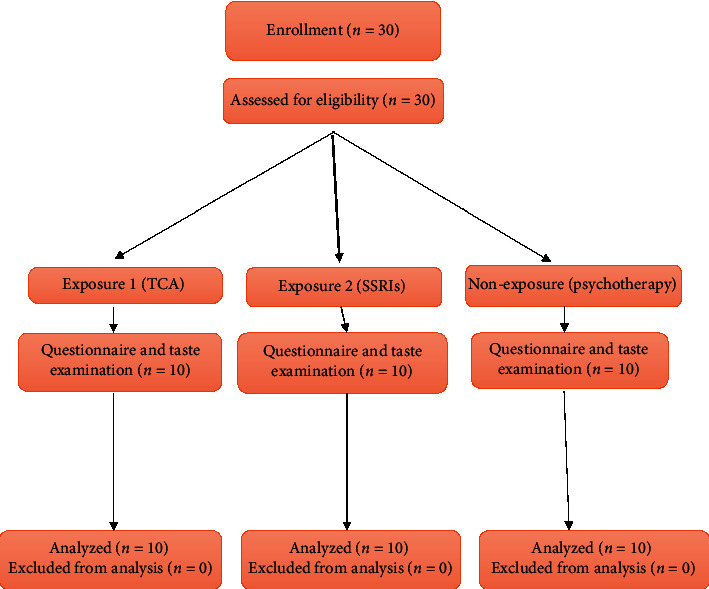
CONSORT flow diagram of the study.

From 30 enrolled patients, 30 patients were included in the study. The flowchart of the patients through the study following the CONSORT flow diagram is presented in Figure 1.

Regarding basic parameters of the studied groups (Table 1), there was no significant difference.

As for the psychiatric disorders which the participants were diagnosed with (Table 1), there was no significant difference between groups for depression alone and depression with other psychiatric problems as obsessive compulsive disorder (OCD) and generalized anxiety disorder (GAD) (*p* = 0.510). Regarding the medical history of the patients, there was no significant difference between groups for systemic diseases (*p* = 0.531) and medications other than antidepressants (*p* = 1.000). Concerning the dental history of the patients, there was no significant difference between groups for dental history including oral hygiene status (*p* = 0.531) and dental care frequency (*p* = 0.406).

The results of the present study revealed that the mean score for sweet detection threshold was 1.4 ± 0.7, 1 ± 0, 1 ± 0 for TCA, SSRI, and psychotherapy, respectively. This was statistically significant (*p* = 0.043). The mean score for sweet detection threshold concentration was 0.9 ± 0.2, 0.8 ± 0, and 0.8 ± 0 for TCA, SSRIs, and psychotherapy, respectively. This was statistically significant (*p* = 0.040). The mean score for recognition threshold of sweet taste was 2.1 ± 1.0, 1.2 ± 0.4, and 1.2 ± 0.4 for TCA, SSRIs, and psychotherapy, respectively. This was statistically significant (*p* = 0.007). The mean score for recognition threshold of sweet taste concentration was 1.2 ± 0.3, 0.9 ± 0.2, and 0.9 ± 0.2 for TCA, SSRIs, and psychotherapy, respectively. This was statistically significant (*p* = 0.008); pairwise comparisons revealed that TCA group was statistically significant from the other 2 groups in sweet taste thresholds (Table 2).

However, in the present study, the mean scores for salt detection threshold (*p* = 0.220), salt detection threshold concentration (*p* = 0.247), recognition threshold of salt taste (*p* = 0.121), and recognition threshold of salt taste concentration (*p* = 0.129) for TCA, SSRIs, and psychotherapy groups were statistically not significant (Table 3). Also, the mean scores for sour detection threshold (*p* = 0.680), sour detection threshold concentration (*p* = 0.572), recognition threshold of sour taste (*p* = 0.880), and recognition threshold of sour taste concentration (*p* = 0.073) for TCA, SSRIs, and psychotherapy were statistically not significant (Table 4). Similarly, the mean scores for bitter detection threshold (*p* = 0.284), bitter detection threshold concentration (*p* = 0.246), recognition threshold of bitter taste (*p* = 0.286), and recognition threshold of bitter taste concentration (*p* = 0.358) for TCA, SSRIs, and psychotherapy groups were statistically not significant (Table 5). These findings indicated that regarding the four taste qualities thresholds, TCA group differed from the other two groups mainly in sweet taste thresholds.

**Figure 2:**
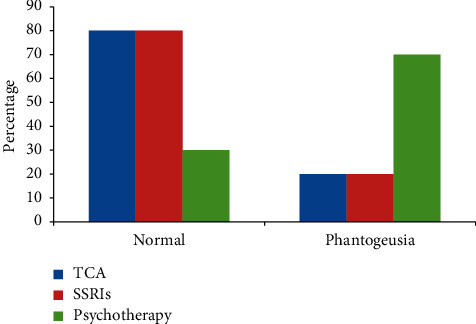
Bar chart representing normal taste and phantogeusia among the studied groups.

Regarding taste abnormalities between groups, the significant difference was detected in the detection of phantogeusia. 70% of psychotherapy group reported phantogeusia, in contrast to only 20% in TCA and SSRIs groups as shown in (Figure 2).

**Figure 3:**
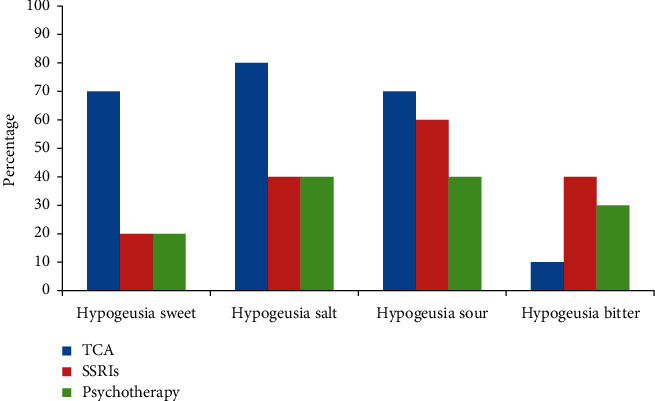
Bar chart representing sweet, salt, sour, and bitter hypogeusia among the studied groups.

Hypogeusia for sweet was statistically significant (*p* = 0.041); 70% of TCA patients suffered from hypogeusia while 20% in SSRI and Psychotherapy group as shown in Figure 3. Abnormalities for other tastes were comparable between groups.

## 4. Discussion

The results of the present study revealed that the mean score for sweet detection threshold was 1.4 ± 0.7, 1 ± 0, 1 ± 0 for TCA, SSRI, and psychotherapy, respectively. This was statistically significant (*p* = 0.043). The mean score for sweet detection threshold concentration was 0.9 ± 0.2, 0.8 ± 0, and 0.8 ± 0 for TCA, SSRIs, and psychotherapy, respectively. This was statistically significant (*p* = 0.040). The mean score for recognition threshold of sweet taste was 2.1 ± 1.0, 1.2 ± 0.4, and 1.2 ± 0.4 for TCA, SSRIs, and psychotherapy, respectively. This was statistically significant (*p* = 0.007). The mean score for recognition threshold of sweet taste concentration was 1.2 ± 0.3, 0.9 ± 0.2, and 0.9 ± 0.2 for TCA, SSRIs, and psychotherapy, respectively. This was statistically significant (*p* = 0.008); pairwise comparisons revealed that TCA group was statistically significant from the other 2 groups in sweet taste thresholds.

However, in the present study, the mean scores for salt detection threshold (*p* = 0.220), salt detection threshold concentration (*p* = 0.247), recognition threshold of salt taste (*p* = 0.121), and recognition threshold of salt taste concentration (*p* = 0.129) for TCA, SSRIs, and psychotherapy groups were statistically not significant. Also, the mean scores for sour detection threshold (*p* = 0.680), sour detection threshold concentration (*p* = 0.572), recognition threshold of sour taste (*p* = 0.880), and recognition threshold of sour taste concentration (*p* = 0.073) for TCA, SSRIs, and psychotherapy were statistically not significant (Table 4). Similarly, the mean scores for bitter detection threshold (*p* = 0.284), bitter detection threshold concentration (*p* = 0.246), recognition threshold of bitter taste (*p* = 0.286), and recognition threshold of bitter taste concentration (*p* = 0.358) for TCA, SSRIs, and psychotherapy groups were statistically not significant. These findings indicated that regarding the four taste qualities thresholds, TCA group differed from the other two groups mainly in sweet taste thresholds.

Regarding taste abnormalities between groups, the significant difference was detected in the detection of phantogeusia. 70% of psychotherapy group reported phantogeusia, in contrast to only 20% in TCA and SSRIs groups. Hypogeusia for sweet was statistically significant (*p* = 0.041); 70% of TCA patients suffered from hypogeusia while 20% in SSRI and Psychotherapy group. Abnormalities for other tastes were comparable between groups.

The results of the present study showed that TCA group was statistically significant from the other 2 groups in sweet detection thresholds scores (*p* = 0.043) and concentrations (*p* = 0.040) and also sweet recognition thresholds scores (*p* = 0.007) and concentrations (*p* = 0.008). This significance could partially be in agreement with Kampov-Polevoy et al. [20, 21] who mentioned that cravings for sweet foods and increased hedonic responses to sweet tastes are described among patients with psychiatric disorders, particularly seasonal affective disorder.

In comparison with previous studies of responses to sweet tastes in Major Depressive Disorders (MDD). Berlin et al. [22] mentioned increased sweet taste thresholds but equivalent hedonic responses to sucrose in adults with and without MDD. Kazes et al. [23] mentioned a preference for sweet foods in MDD that was not changed with antidepressant therapy, but responses to varying concentrations of sweet solutions were not officially assessed, in which the present study was partially in contrast with it.

However, there was a contradiction to the above mentioned studies and the present study in a study by Dichter et al. [24] to examine responses to sweet tastes via the Sweet Taste Test (STT [25]) in adult outpatients with MDD before and after psychotherapy and compared response profiles to those of nondepressed control participants also examined at two points in time, in which they reported that there were no significant differences in STT response profiles between groups overall or at either time point and did not differ after psychotherapy, relative to baseline. These findings suggested that although anhedonia may be a symptom of MDD, the disorder was not characterized by altered responses to sweet tastes.

Concerning SSRIs group in the present study, clinical reports mentioned that some patients associate SSRIs treatment with an experience of emotional blunting through which emotional responses to both aversive and pleasurable experiences are decreased [26, 27]. Experimental studies in animals and humans indicated that serotonin pathways exhibited an inhibitory impact over neural systems mediating both positive and negative affective processes [28]. Thus, increasing the serotonin function by SSRIs would produce a form of “emotional constraint” in which the salience of both rewarding and aversive stimuli is lost [28]. These findings could be in partial agreement with the present study where TCA group was found to be statistically significant from SSRIs group in sweet taste detection and recognition thresholds, but it could not be denied that there were still greater sweet taste thresholds among SSRIs group which meant that the salience of rewarding was not lost completely. However, evaluating the effect of SSRIs on emotional responses in depressed patients is difficult as modest degrees of emotional blunting might be difficult for individuals to detect or report subjectively [29].

Considering TCA group, it was found to be statistically significant from the other 2 groups in sweet taste thresholds in the present study. This finding was in agreement with Maddox et al. [30] who reported that the taste side effects of tricyclic antidepressants reported would contribute to the discontinuation rates reported in studies of depressed patients. It was also concluded that the discontinuation rate due to side effects was higher for tricyclic antidepressants than for selective serotonin reuptake inhibitors by Anderson and Tomenson and Martin et al. [31, 32].

Moreover, in a randomized controlled study by Ramzy [19], the combined use of pregabalin plus paroxetine (SSRI) for fibromyalgia management in comparison with the combined use of pregabalin plus either amitriptyline (TCA) or venlafaxine was evaluated. Medication termination due to drowsiness, dizziness, blurred vision, abnormal taste, hunger, hallucination, urination problems, and sexual dysfunction was reported mostly in the amitriptyline group. It was concluded that the combined use of pregabalin plus paroxetine provides an effective method with increased tolerability to decrease the somatic and depressive symptoms of fibromyalgia and to improve the quality of life in affected individuals. These findings supported that TCA had more taste side effects and less tolerability than SSRIs which supported the revealed result of the present study concerning TCA group being statistically significant from the other 2 groups in sweet taste thresholds.

However, in the present study, the mean scores for salt detection threshold, salt detection threshold concentration, recognition threshold of salt taste, and recognition threshold of salt taste concentration for TCA, SSRIs, and psychotherapy groups were statistically not significant (*p* = 0.220), (*p* = 0.247), (*p* = 0.121), and (*p* = 0.129), respectively. Also, the mean scores for sour detection threshold, sour detection threshold concentration, recognition threshold of sour taste and recognition threshold of sour taste concentration for TCA, SSRIs, and Psychotherapy were statistically not significant (*p* = 0.680), (*p* = 0.572), (*p* = 0.880), and (*p* = 0.073), respectively. Similarly, the mean scores for bitter detection threshold, bitter detection threshold concentration, recognition threshold of bitter taste, and recognition threshold of bitter taste concentration for TCA, SSRIs, and psychotherapy groups were statistically not significant (*p* = 0.284), (*p* = 0.246), (*p* = 0.286), and (*p* = 0.358), respectively. These findings indicated that regarding the four taste qualities taste thresholds, that the most affected taste is sweet taste thresholds and TCA group differed from the other two groups particularly in sweet taste thresholds. These findings were in contrast to a study performed by Schiffman et al. [33], who reported that intermittent application of the tricyclic antidepressant amitriptyline HCl led to taste decrements only for salts, while continuous application affected all of the taste qualities to varying degrees.

Considering studies performed on depressive patients, it would be essential to separate the possible effect of depressive status on taste responses from systemic use of antidepressant medications as it has been known that depressive symptomology affects scores on cognitive measures, and emotional factors may impact sensory/perceptual responses also. Therefore, the present study included a nonexposure group which included depressed patients under nonpharmacological therapy.

The present study revealed a significant difference between groups that was detected in phantogeusia (*p* = 0.041), where 70% of psychotherapy group reported phantogeusia, while only 20% in TCA and SSRIs groups which is considered in agreement with Dess and Edelheit [34] who found that subjects would particularly rate stimuli as more bitter if they were placed under stress or [35] scored higher on subclinical depression scores.

Moreover, clinical reports mentioned that antidepressant drugs could cause taste disturbances including dysgeusia (distortion of taste), hypogeusia (diminished sensitivity of taste), and ageusia (absence of taste) [36] which is in acceptance with the present study which revealed that hypogeusia for sweet was statistically significant (*p* = 0.041), where 70% of TCA patients had hypogeusia while only 20% in both SSRI and psychotherapy groups. This is also in agreement with Schiffman et al. [14] who reported that both first-generation tricyclic antidepressants and later-developed selective serotonin reuptake inhibitors are mentioned clinically to cause chemosensory complaints. However, McClain et al. [37] reported that hypogeusia is common in acutely ill patients and those recovering from prolonged illness. It is an incidental finding as patients generally do not complain of it. It is only discovered in discussions on condiments of food or salt intake. A number of diseases such as rheumatoid arthritis, Crohn's disease, trauma, stress, and thermal injury that lead to marked losses of zinc, resulting in hypogeusia.

The oral health should not be separated from the remainder of the body as it affects general health by causing considerable pain and by changing what people eat, their speech, and their quality of life and well-being. Gustatory dysfunction should not be neglected in depressed patients and those under antidepressants therapy. It should be considered as a real pathophysiological symptom in depressed patients, and more work should be done on the mechanisms through which gustatory function is disturbed in depression. More experimental studies should be done to essentially quantify potential taste changes among patients under antidepressants therapy.

Health care providers should know the causes of taste disorders in order to be capable of reaching correct diagnosis and planning treatment without unnecessary dental treatment. It should be noted that before taste changes assessment for any causes, dental causes should be first ruled out and the diagnosis of a gustatory dysfunction must be supported with a thorough medical history, the patient's subjective reporting, and psychophysical testing.

## 5. Conclusions

The results of the present study can conclude that gustatory dysfunction is a neglected system among the depressed patients and those under antidepressants therapy till now, where gustatory dysfunction was found to be mostly associated with TCA followed by SSRIs and the depressed patients undergoing psychotherapy particularly for sweet taste thresholds. Moreover, phantogeusia was common among depressed patients undergoing psychotherapy while hypogeusia for sweet was more common among TCA than SSRIs. More attention has to be given to taste changes among these patients as oral health affects general health by causing considerable pain and by changing what people eat, their speech, and their quality of life and wellbeing. Proper awareness and evaluation of this problem will reduce its impact and improve the quality of life for the depressed patients.

## Figures and Tables

**Table 1:** Descriptive statistics, one-way ANOVA, Chi square tests, and fisher's exact test for the demographic data and other variables of the tested groups.

Variables		Group						
		TCA (*n* = 10)		SSRIs (*n* = 10)		Psychotherapy (*n* = 10)		*p* value
		No.	%	No.	%	No.	%	
Age (yrs.)	Mean ± SD	38.6 ± 7.6		35.7 ± 8.7		29.9 ± 8.2		0.072
	Range	24–50		22–47		21–50		

Sex	Female	8	80.0	6	60.0	8	80.0	0.668
	Male	2	20.0	4	40.0	2	20.0	

Weight (kg)	Mean ± SD	78.1 ± 7.8		74.8 ± 21.8		63.6 ± 10.4		0.087
	Range	65–90		45–100		46–75		

Residence	Rural	2	20.0	4	40.0	0	0	0.082^a^
	Urban	8	80.0	6	60.0	10	100.0	

Marital status	Divorced	0	0.0	0	0.0	1	10.0	NA
	Married	3	30.0	5	50.0	4	40.0	
	Single	5	50.0	5	50.0	5	50.0	
	Widow	2	20.0	0	0.0	0	0.0	

Marital status	Married	3	30.0	5	50.0	5	50.0	0.527
	Single	5	50.0	5	50.0	5	50.0	
	Widow	2	20.0	0	0.0	0	0.0	

Education	Basic/no education	4	40.0	5	50.0	0	0.0	0.073^a^
	Secondary/high school	2	20.0	3	30.0	3	30.0	
	University degree	4	40.0	2	20.0	7	70.0	

Work status	Not working	6	60.0	6	60.0	8	80.0	0.698^a^
	Working	4	40.0	4	40.0	2	20.0	
	Yes	1	10.0	3	30.0	2	20.0	

Depression comorbid	Depression alone	8	80.0	6	60.0	5	50.0	0.510
	With other psychiatric problems	2	20.0	4	40.0	5	50.0	

Systemic diseases	No	7	70.0	4	40.0	6	60.0	0.531
	Yes	3	30.0	6	60.0	4	40.0	

Medication	No	6	60.0	5	50.0	6	60.0	1.000
	Yes	4	40.0	5	50.0	4	40.0	

Oral hygiene status	Bad	7	70.0	6	60.0	4	40.0	0.531
	Good-moderate	3	30.0	4	40.0	6	60.0	

Dental care frequency	Irregular	3	30.0	5	50.0	5	50.0	0.406
	Regular	0	0.0	1	10.0	2	20.0	
	Symptomatic	7	70.0	4	40.0	3	30.0	

**Table 2:** Descriptive statistics and one-way ANOVA for sweet detection and recognition thresholds scores and concentrations among the tested groups.

		Mean	SD	95% CI		Min.	Max.	*p* value
				Lower	Upper			
Sweet detection threshold score	TCA^∗^	1.4	0.7	0.9	1.9	1.0	3.0	0.043
	SSRIs	1.0	0.0	1.0	1.0	1.0	1.0
	Psychotherapy	1.0	0.0	1.0	1.0	1.0	1.0

Sweet detection threshold conc	TCA^∗^	0.9	0.2	0.8	1.1	0.8	1.4	0.040
	SSRIs	0.8	0.0	0.8	0.8	0.8	0.8
	Psychotherapy	0.8	0.0	0.8	0.8	0.8	0.8

Sweet recognition threshold score	TCA^∗^	2.1	1.0	1.4	2.8	1.0	4.0	0.007
	SSRIs	1.2	0.4	0.9	1.5	1.0	2.0
	Psychotherapy	1.2	0.4	0.9	1.5	1.0	2.0

Sweet recognition threshold conc	TCA^∗^	1.2	0.3	1.0	1.4	0.8	1.6	0.008
	SSRIs	0.9	0.2	0.8	1.0	0.8	1.2
	Psychotherapy	0.9	0.2	0.8	1.0	0.8	1.2
	SSRIs	3.7	2.1	2.2	5.2	1.0	6.0
	Psychotherapy	5.1	1.7	3.9	6.3	2.0	6.0

**Table 3:** Descriptive statistics and one-way ANOVA for salt detection and recognition thresholds scores and concentrations among the tested groups.

		Mean	SD	95% CI		Min.	Max.	*p* value
				Lower	Upper			
Salty_Detection threshold _score	TCA	1.9	0.9	1.3	2.5	1.0	3.0	0.220
	SSRIs	1.4	0.8	0.8	2.0	1.0	3.0	
	Psychotherapy	1.3	0.7	0.8	1.8	1.0	3.0	

Salty_Detection threshold _ conc	TCA	0.1	0.1	0.0	0.2	0.0	0.2	0.247
	SSRIs	0.1	0.1	0.0	0.1	0.0	0.2	
	Psychotherapy	0.0	0.1	0.0	0.1	0.0	0.2	

Recognition threshold of Salty taste_score	TCA	2.7	1.2	1.9	3.5	1.0	4.0	0.121
	SSRIs	1.8	1.2	0.9	2.7	1.0	4.0	
	Psychotherapy	1.7	1.1	0.9	2.5	1.0	4.0	

Recognition threshold of Salty taste_conc	TCA	0.2	0.1	0.1	0.3	0.0	0.3	0.129
	SSRIs	0.1	0.1	0.0	0.2	0.0	0.3	
	Psychotherapy	0.1	0.1	0.0	0.2	0.0	0.3	

**Table 4:** Descriptive statistics and one-way ANOVA for sour detection and recognition thresholds scores and concentrations among the tested groups.

		Mean	SD	95% CI		Min.	Max.	*p* value
				Lower	Upper			
Sour taste_Detection threshold _score	TCA	2.9	1.4	1.9	3.9	1.0	5.0	0.680
	SSRIs	2.4	1.8	1.1	3.7	1.0	5.0	
	Psychotherapy	3.0	1.6	1.8	4.2	1.0	5.0	

Sour taste_Detection threshold _ conc	TCA	0.2	0.4	0.1	0.4	0.0	1.2	0.572
	SSRIs	0.4	0.6	0.0	0.8	0.0	1.2	
	Psychotherapy	0.4	0.6	0.0	0.8	0.0	1.2	

Recognition threshold of Sour taste_score	TCA	3.7	1.7	2.5	4.9	1.0	6.0	0.880
	SSRIs	3.3	1.9	1.9	4.7	1.0	6.0	
	Psychotherapy	3.7	2.2	2.0	5.3	1.0	6.0	

Recognition threshold of Sour taste_conc	TCA	0.4	0.6	0.0	0.9	0.0	1.2	0.073
	SSRIs	0.0	0.0	0.0	0.0	0.0	0.1	
	Psychotherapy	0.1	0.0	0.0	0.1	0.0	0.1	

**Table 5:** Descriptive statistics and one-way ANOVA for bitter detection and recognition thresholds scores and concentrations among the tested groups.

		Mean	SD	95% CI		Min.	Max.	*p* value
				Lower	Upper			
Bitter taste_Detection threshold _score	TCA	3.9	1.8	2.6	5.2	1.0	5.0	0.284
	SSRIs	2.9	1.9	1.6	4.2	1.0	5.0	
	Psychotherapy	4.1	1.7	2.9	5.3	1.0	5.0	

Bitter taste_Detection threshold _ conc	TCA	0.7	0.4	0.4	0.9	0.0	0.9	0.246
	SSRIs	0.4	0.4	0.1	0.7	0.0	0.9	
	Psychotherapy	0.7	0.4	0.4	1.0	0.0	0.9	

Recognition threshold of Bitter taste_score	TCA	4.7	2.2	3.2	6.3	1.0	6.0	0.286
	SSRIs	3.7	2.1	2.2	5.2	1.0	6.0	
	Psychotherapy	5.1	1.7	3.9	6.3	2.0	6.0	

Recognition threshold of Bitter taste_conc	TCA	0.1	0.2	−0.2	0.5	0.0	0.3	0.358
	SSRIs	0.2	0.1	0.1	0.3	0.0	0.3	
	Psychotherapy	0.4	0.4	−0.7	1.5	0.2	0.9	

## Data Availability

The data used to support the findings of this study are included within the article.
